# Is serum Interleukin-17 associated with early atherosclerosis in obese patients?

**DOI:** 10.1186/s12967-014-0214-1

**Published:** 2014-08-06

**Authors:** Giovanni Tarantino, Susan Costantini, Carmine Finelli, Francesca Capone, Eliana Guerriero, Nicolina La Sala, Saverio Gioia, Giuseppe Castello

**Affiliations:** Department of Clinical Medicine and Surgery, Federico II University Medical School of Naples, Via Sergio Pansini 5, 80131 Naples, Italy; Centro Ricerche Oncologiche di Mercogliano, Istituto Nazionale Per Lo Studio E La Cura Dei Tumori “Fondazione Giovanni Pascale”, IRCCS, 83013 Mercogliano, (Av) Italy; Center of Obesity and Eating Disorders, Stella Maris Mediterraneum Foundation, C/da S. Lucia, Chiaromonte, 80035 Potenza, Italy

## Abstract

Atherosclerosis is a chronic inflammatory process of the vessel walls, and CD4^+^ T-cells are peculiar to both human and murine atherosclerotic lesions. There is a recent line of research favoring hypothetic allergic mechanisms in the genesis of atherosclerosis and, consequently, coronary artery disease (CAD), among which Interleukin (IL)-17 appears to be a key cytokine regulating local tissue inflammation.

The objective was to add a piece of information on the role of IL-17 in the genesis of atherosclerosis. Eighty obese patients with normal liver enzyme levels but presenting with ultrasonographic evidence of NAFLD formed the population of this cross-sectional study. Anthropometric measures, data on excess adiposity, metabolic profile, serum concentrations of IL-17, eotaxin-3, IL-8, and CCL4/MIP1β, C-reactive protein, fibrinogen, ferritin, TNF-α, as well carotid intima-media thickness (IMT), a marker of atherosclerosis, and the main risk factors for CAD, such as blood pressure and smoking status, but also less determinant ones such as degree of NAFLD severity, Intramuscular Triglyceride storage and Resting Metabolic Rate were evaluated.

Serum concentrations of Il-17 were detected as related to those of inflammatory cytokines, IL-6, IFN-γ and TNF-α. Furthermore, circulating levels of IL-17 were linked to those mirroring allergic process, IL-8, CCL4/MIP1β and eotaxin. Early atherosclerosis, evidenced as increased IMT, was not associated with circulating IL-17 levels. At multiple regression, IMT was predicted, other than by age, by the amount of the visceral adiposity, expressed as visceral adipose tissue at ultrasonography, and by serum eotaxin.

In conclusion, a strong relationship was found between the IL-17-related chemokine eotaxin and IMT. The association found between the amount of visceral fat and circulating levels of eotaxin on the one hand, and IMT on the other, could reinforce the hypothesis that IL-17, released by the visceral adipose tissue, induces eotaxin secretion via the smooth muscle cells present in the atheromatosus vessels.

## Introduction

Metabolic Syndrome (MS) and obesity, mainly visceral obesity, are associated with increased mortality due to Coronary Artery Disease (CAD). Common carotid Intima-Media Thickness (IMT) is a functional and structural marker of the atherosclerotic process that leads to CAD [1]. However, also unclassified Non-Alcoholic Fatty Liver Disease (NAFLD) or Hepatic Steatosis (non-alcoholic HS), a further expression of the MS [2], is associated with increased CAD risk [3]. Resting Metabolic Rate (RMR), by showing an obesity-induced reduced energy generation for myocardial contractile function, is a novel biomarker of CAD risk [4]. Intramuscular Triglyceride (TG) storage (ImTG), easily evaluated by UltraSound due to an increase in muscle echo intensity [5], was associated with metabolic risk factors, although most of these associations were lost after adjustment for BMI or visceral adipose tissue (VAT), [6].

Starting from the evidence that atherosclerosis is a chronic inflammatory process of the vessel walls and that CD4^+^ T-cells are peculiar to both human and murine atherosclerotic lesions [7,8], there is a recent line of research favoring hypothetic allergic mechanisms in the genesis of atherosclerosis and, consequently, CAD [9,10]. The main function of Interleukin (IL)-17 appears to be the regulation of local tissue inflammation via the coordinated expression of pro-inflammatory cytokines and chemokines. This cytokine is primarily involved in the pathogenesis of allergic diseases [11]. The relevance of IL-17 to human atherosclerosis remains poorly defined because of the conflicting results available; what is more, the underlying mechanisms have yet to be unraveled. Results from animal studies suggest a pro-atherogenic role for IL-17 [12,13]; in humans, vice versa, an athero-protective effect of IL-17, through the cross-regulation of IFN-γ-producing Th1 cells, has been recently proposed [14]. Accordingly, Simon et al. have found an inverse association between levels of circulating IL-17 and a higher risk of major cardiovascular events [15].

A recent research has been conducted to assess IL-17-related cytokine/chemokine interplay using a different target-organ i.e., cultured bronchial epithelial cells; these authors demonstrated that treating these cells with IL-17 plus T(H)2 cytokines induced a strong up-regulation of IL-8, eotaxin-3 and CCL4/MIP1β [16].

IL-17-induced IL-8, as discussed below, is a cytokine with a pleiotropic effect on cardiovascular homeostasis. On the other hand, IFN-γ inducible protein, CXCL10/IP-10, a member of the chemokine family with pro-inflammatory and anti-angiogenic properties, has been proposed as a key link between inflammation and angiogenesis [17], lending credence to its role in inducing atherosclerosis.

Zeroing in on IMT, we aimed at evaluating the associations between this marker of atherosclerosis and CAD risk factors in our study population.

Moreover, to gain further insight into the role of IL-17 in the genesis of atherosclerosis, the serum concentrations of IL-17 were correlated, beyond IMT, to established CAD risk factors, i.e., blood pressure, smoking status, High Density Lipoprotein cholesterol (HDL) and Low Density Lipoprotein cholesterol (LDL), but also less determinant CAD risk factors such as metabolic fuel utilization, evaluated as RMR, new parameters positively associated with subclinical carotid atherosclerosis in overweight/obese individuals [18], ImTG and NAFLD; fat excess distribution; metabolic profile; markers of acute and chronic inflammation; cytokine/chemokine network generated by IL-17, such as IL-8, CXCL10/IP-10, CCL4/MIP1β, IFN-γ and eotaxin.

## Methods

### Study design and population

Our study sample consisted of 125 consecutive obese adults with NAFLD, without known CAD, referred to the out-patient metabolic unit from May 2012 to April 2013, all diagnosed at least six months before enrollment. The subjects had been on a balanced low calorie, low fat (25% of calories) diet for three months prior to enrolment and were characterized by sedentary life-style. The research protocols were approved by the Ethics Committee of the Federico II University Medical School of Naples (assigned protocol number: 231-05). All participants provided their written informed consent to participate in this study. The Department of Clinical Medicine and Surgery of the Federico II University Medical School of Naples (Italy) approved the conduction of this study.

For the current analysis, we used residual serum from a subsample of 80 patients, median age 46 years, recruited during the same period, who had participated in the parent study on Eotaxin and had agreed to contribute to this second part. Standard checklist form, which included information on age, sex, diet, lifestyle, past or present disease(s), current medications and dosage, self-reported tobacco products usage (cigarettes brand and type, cigars, or tobacco for pipes, used in the days before enrolment, and self-reported tobacco exposure (presence or absence of smokers at home, at work, or in social settings).

Detailed descriptions of the inclusion and exclusion criteria of the study design and data collection procedures have been described elsewhere [10]. Briefly, out of the initial participants, 12 were excluded due to difficulties in performing abdominal US for NAFLD screening (intestinal meteorism), nine others because they had taken steroids (six for bronchial asthma, two for rheumatoid arthritis and one for IBD), and eight who had undergone therapy with one or more drugs known to alter laboratory data such as aspirin, statins, fibrates and metformin. Six patients were excluded for the presence of hepatic co-morbidities in their history (HBV/HCV infection or alcohol abuse). Ten patients were excluded for lack of adherence to the protocol. Every patient gave informed written consent to participate to this investigation according to the principles laid down in the declaration of Helsinki.

### Evaluation of excess adiposity

The three degrees of obesity (light, moderate and severe) were established on the basis of BMI cut-off points of 30–34.9, 35–39.9 and >40 kg/m^2^, respectively. MS was not categorized as a single entity, but analyzing every criterion. Visceral obesity was identified by measuring WC at the midpoint between the lower border of the rib cage and the iliac crest. Hip circumference was measured around the widest part of the buttocks, with the tape parallel to the floor, and the Waist to Hip ratio (WHR) was calculated.

Subcutaneous Adipose Tissue (SAT) and Visceral Adipose Tissue (VAT) were assessed by transverse scanning using an eleven linear probe and 3.5 MHz convex probe, respectively. SAT was defined as the thickness between the skin-fat interface and the linea alba, avoiding compression. VAT was defined as the distance between the anterior wall of the aorta and the internal face of the recto-abdominal muscle perpendicular to the aorta, measured one cm above the umbilicus. When the aortic walls were not visualized because obscured by bowel gas, the Doppler scan was used.

The classification of “bright liver” or HS was based on the following scale of hyper-echogenity: grade 0 = absent, grade 1 = light, grade 2 = moderate, grade 3 = severe, pointing out the difference between liver densities and the right kidney [19]. Mean brightness levels of both liver and right kidney cortex were obtained on the same longitudinal sonographic plane.

Muscle ultrasound, performed at the level of the biceps muscle of the left superior arm, is a convenient technique to visualize pathological muscle tissue, as it is non-invasive and provides results in real-time. Both infiltration of fat and fibrous tissue increase muscle echo intensity, i.e., the muscles become whiter at the ultrasound image [20]. To describe muscle echo intensity, Heckmatt and co-workers developed a visual grading scale where grade I represented normal muscle and grade IV a severely increased muscle echo intensity with total loss of bone echo (we chose biceps versus humerus), [21]. The levels of brightness of the liver and the biceps were calculated three times directly from the frozen images.

### Atherosclerosis and caronary artery disease risk factors

The common carotid, the carotid bulb and the near and far wall segments of the internal carotid were scanned bilaterally. Subjects were examined in the supine position with the head turned 45° contra-lateral to the side undergoing scanning. Images were obtained in longitudinal section, with a single lateral angle of isonation, optimizing the image for the far wall. IMT was defined as the distance between the lumen-intima and the media-adventitia ultrasound interfaces. Measurements were performed off-line and consisted of six manual measurements at equal distances along 1 cm on the far wall of the common carotid. Left and right IMT were averaged [22].

Systolic/Diastolic Arterial Pressure (SBP, DBP) was the average of three consecutive readings taken after allowing the subjects to rest for five minutes in the sitting position.

RMR was measured by indirect calorimetry using a canopy system (V max 29 N, Sensor Medics, Anaheim, USA) in a quiet environment and with patients in the supine position for 30 min before measurement. After a 15–20 min adaptation to the instrument, oxygen consumption and carbon dioxide production were determined for 45 min. Energy expenditure was derived from CO2 production and O2 consumption with the appropriate Weir formula neglecting protein oxidation [23]. BMR, expressed as kcal/24 h, was adjusted for changes in fat-free mass (FFM)], which was evaluated by single-frequency bio-impedance analysis obtaining an RMR/FFM ratio, expressed as kcal/24 h*kg of body [24].

Age and smoking status were also considered important risk factors.

### Inflammatory markers

The methods used to assess circulating IL-6 and TNF-α are described below. C reactive protein (CRP) values were determined by a high-sensitivity ELISA test, with reference values between 0.3 and 8.6 mg/L in healthy men and between 0.2 and 9.1 mg/L in healthy women (BioCheck, Inc, CA, USA). Ferritin and fibrinogen were performed by in-house standard procedures.

### Metabolic profile

Serum Triglycerides (TG), HDL, LDL, basal insulin, were determined by standard methods. IR status was determined by the HOmeostatic Metabolic Assessment (HOMA), which was assessed by the following formula: fasting insulin (μU/mL) x fasting glucose (mg/dL)/405. Moreover, as the repeated HOMA measurements presented high within-person variability in obese patients, HOMA values were averaged on the basis of several determinations to avoid misclassification.

### Bead-based assay

Human IL-17 singleplex was performed according to Bio-Rad systems protocol (Bio-Rad Lab., Inc., Hercules, CA, USA) as elsewhere reported [25,26]. Sera samples were diluted four times with a suitable buffer. Initially, the 96-well filter bottom plate was pre-wet. Fifty microliters of diluted microparticle solution and 50 μl of sample were added to each well in duplicate. Thereafter, the plate was incubated for 1 h and washed three times with wash buffer. Afterwards, 25 μl of diluted biotin antibody were added to each well and incubated for 1 h. The plate was then washed as described above, and 50 μl of diluted Streptavidin-PE were added to each well and incubated for 10 min. All incubations were performed at room temperature on an orbital shaker set at 15 g. Finally, the plate was washed again with 100 μl of wash buffer. The median relative fluorescence units were measured using the Luminex 200 analyzer (Luminex, Austin, TX, USA). IL-17 concentrations were calculated using a standard curve. Control range used for IL-17 was 52,89 ± 41,79 pg/mL.

The coefficient of variation, calculated by SD/mean × 100 for the intra-assay and inter-assay was <10% and <12%, respectively.

To establish whether IL-17 levels correlated with other cytokines, the following molecules present in Human Cytokine 27-Plex Panel (Bio-Rad Lab., Inc., Hercules, CA, USA), i.e., IL-1β, IL-1ra, IL-2, IL-4, IL-5, IL-6, IL-7, IL-8, IL-9, IL-10, IL-12 (p70), IL-13, IL-15, eotaxin (CCL11), basic FGF, G-CSF, GMCSF, IFN-γ, IP-10 (CXCL10), MCP-1 (CCL2), MIP-1a (CCL3), MIP-1β (CCL4), PDGF-bb, RANTES (CCL5), TNF- α and VEGF, were evaluated by the same procedure reported above, the control range of which was: for IL-17: 52.89+/−41.79 pg/mL, eotaxin 19.4+/−7.2 pg/mL, IL-8: 18.04+/−2.09 pg/mL, IP-10 (CXCL10): 242.09+/−52.67 pg/mL, CCL4/ MIP-1β: 22.51+/−9.65 pg/mL, IFN-γ: 19.79+/−3.26 pg/mL, for IL-6: 4.01+/−2.58 pg/mL, for TNF-α : 5.76+/−3.44 pg/mL.

### Remainders

Blood samples were drawn into 9-mL serum tubes, with participants in the fasting state. Samples were centrifuged for 10 min at RCF of 850–1000, collected, aliquoted, and frozen at −20°C until analysis. Alanine aminoTransferase (ALT), Pseudo Cholinesterase (PCH), Alkaline Phosphatase (AP), Gamma Glutamyl Transpeptidase (gamma-GT) were obtained following standard procedures.

### Statistics

Age, BMI, WHR, SAT, VAT, ALT, AP, γ-GT, HOMA, SBP, DBP, common carotid IMT, TG, CRP, fibrinogen, ferritin, eotaxin, fasting glucose, IFN-γ, IP-10 (CXCL10), MIP-1β (CCL4), IL-17, IL-8, IL-6, TNF- α were not normally distributed when analyzed by the Shapiro-Wilk (S-W) test, p < 0.05, and were expressed as median plus 25–75 inter-quartile range (IQR). Data for WC, CHE, HDL cholesterol, derived from a normally distributed population (S-W, p > 0.05), were articulated as mean plus SD. Smoking status, categorized as present/absent, was analyzed in relation to its role influencing IMT by means of the two-tailed Mann–Whitney test for independent samples, expressed as median plus 95% CI for the median. At univariate analysis, to assess the independent effect of a quantitative variable on the prediction of another one, the linear regression analysis (least squares) was used, evaluating the coefficient with its standard error, 95% confidence intervals (CI) and t (t-value). A t-value above 1.96 with a significance below 0.05 indicates that the independent variable is a significant predictor of the dependent variable within and beyond the sample. To establish the best combination of independent (predictor) variables able to predict the dependent (predicted) variable, multiple regression (Stepwise Selection) was adopted firstly entering all independent variables *if p = <0.05* into the model, and then removing *if p= >0.1* the non-significant variables sequentially, with a maximum number of 10 steps; this produced two different analyses, i.e., IL-17 or IMT used as dependent variable, in the first and second model, respectively. To avoid multi-collinearity, i.e., situations in which the predictors are correlated with each other to some degree, the variance inflation factor and tolerance were set at >10 and <0.1, respectively. Similarly, to get the sense of which variables contribute more or less to the regression equation, the magnitude of standardized coefficient beta (β) was calculated. Finally the zero order correlation coefficients, indicating the simple correlation coefficients, were evaluated.

To evaluate intra/inter-observer variability of the measurements, the mean difference in the measurements of the observers was first calculated. Next, the concordance correlation coefficient (ρ_c_), which measures precision and accuracy, was adopted to evaluate the degree of pair observations at US, with values >0.8 considered as indicators of good reliability. MedCalc, version 13.2 (MedCalc Software, Broekstraat 52, 9030 Mariakerke, Belgium) and SyStat 13 (Cranes Software International, Bangalore, India) were the packages used for the statistics.

## Results

Demographic characteristics, anthropometric parameters/US features evaluating excess adiposity, metabolic profile, inflammatory markers, IMT and coronary artery disease risk factors are reported in Table 1. This clearly shows that patients were affected mainly by II and III degree obesity; some patients (n 11, 13%) did not present ImTG and most of them were characterized by mild/moderate HS (n 60, 75%). Noteworthy are the normal or slightly elevated values of liver enzymes in these obese patients, who nevertheless presented liver fat storage excess. Finally, the metabolic profile, the borderline median IMT and blood pressure values of our series were not particularly altered, showing that they were selected to study a homogeneous population without unbalanced CAD risks. Thirty-two of 80 patients enrolled reported to be active smokers, and ten passive smokers. Twenty-two subjects had never smoked, , and 36 were past smokers. IMT did not differ in the two groups , i.e., 0.09 (0.08-0.11) and 0.08 (0.08-0.10) median plus (95% CI of median), respectively, P = 0.60. Among the cytokine/chemokine network, our data highlighted a significant increase in circulating TNF-α, Eotaxin, IL-8 and IFN *γ* levels, whereas serum IL-6 and CCL4/MIP1β were slightly elevated.

**Table 1:** **Characteristics of obese patients with NAFLD (n 80)**

**Parameter**	**Mean+/−SD or median plus (25–75 IQR)**	**Parameter**	**Mean+/−SD or median plus (25–75 IQR)**
Age (years)	46 (34–53)	HDL Females mg/dL	49.3+/−15
Gender M/F	36/44	HDL Males mg/dL	42.7+/−9
Obesity Degree I/II/III (n)	18/26/46	TG mg/dL	123.5 (83.5-188)
BMI	42.3 (38.1-46.8)	CRP mg/mL	0.56 (0.27-1.3)
WC Females (cm)	118.9+/−12.5	Fibrinogen g/L	295.5 (256–357.5)
WC Males (cm)	129.3+/−14	Ferritin Females ng/mL	41.5 (20–69)
WHR Females	0.95 (0.93-0.97	Ferritin Males ng/mL	167.5 (85–234)
WHR Males	0.98 (0.96-1)	SLD cm	11.3+/−1.5
HS Grade 1/2/3 at US (n)	22/48/10	RMR	2352.2+/−432.7
SAT (cm)	2.6 (2.1-3.1)	Eotaxin pg/mL	24.6 (6.17-77.5)
VAT (cm)	7.5 (6–9.4)	TNF- α pg/mL	41.8 (8.1-112.7)
ImTG Score I/II/III/IV at US(n)	11/23/30/16	IL-6 pg/mL	5.7 (2.3-17.5)
ALT (U/L)	28 (21.5-29)	IL-8 pg/mL	23.1 (3.3-68.1)
CHE (U/L)	9671.4+/−1882.2	IFN-γ pg/mL	158 (56–390)
AP (U/L)	73 (61–91)	IP-10 (CXCL10) pg/mL	405 (255–659)
γ-GT(U/L)	25 (16.5-42.5)	MIP-1β (CCL4) pg/mL	37.2 (24.3-51.9)
HS grade at US 1/2/3 (n)	22/50/8	Fasting insulin μU/mL	10.9 (7.5-15.8)
IMT (mm)	0.09 (0.07-011)	Fasting glucose mg/dL	96.5 (87–114)
SBP mm Hg	130 (120–140)	HOMA	2.78 (1.85-4.18)
DBP mm Hg	80 (80–90)	LDL	66.8 (33.3-140)
Smoking status (yes/no)	42/38		

Waist Circumference WC, Body Mass Index BMI, Waist to Hip ratio WHR, Subcutaneous Adipose Tissue SAT, Visceral Adipose Tissue VAT, High Density Lipoprotein-cholesterol HDL, Low Density Lipoprotein LDL, TriGlycerides TG, C Reactive Protein CRP, Spleen Longitudinal Diameter SLD, Tumor Necrosis Factor alpha TNF-α*,* Interleukin-6 IL-6, Interleukin-8 IL-8, C-X-C motif chemokine 10 (CXCL10) also known as Interferon Gamma-induced protein 10 (IP-10) CXCL10/IP-10, Chemokine (C-C motif) ligand 4 CCL4/MIP1β, Interferon-Gamma IFN-γ UltraSound US, ALanine aminoTransferase ALT, CHolinEsterase CHE, Alkaline Phosphatase AP, Gamma-Glutamyl Transglutaminase γ-GT, fasting glucose, High Density Lipoprotein cholesterol HDL, HOmeostatic Metabolic Assessment HOMA, Systolic Blood Pressure SPP, Diastolic Blood Pressure DBP, Intima-Media Thickness IMT Resting Metabolic Rate RMR, Intramuscular Triglyceride (TG) storage Score ImTG.

The intra/inter-observational variability of the US estimations was not significant, the mean difference being equal to 1.7, 2.2, 2.5, 2.3 and 1.9%, and 2.1, 3.3, 3.9, 4.6, and 3. 1% for the HS, VAT, SAT, SLD and common carotid IMT, respectively, with a ρ_c_ of 0.92.

### Predictions

**Figure 1:**
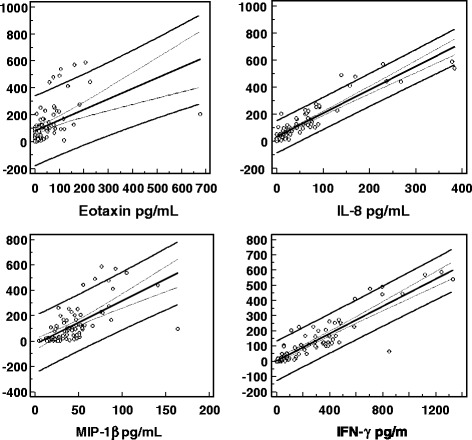
**Graphics of the main predictions between IL-17 and other immune parameters.** At the centre the regression line is evidenced; 95% confidence interval as dot line and 95% prediction interval as continuous line, Chemokine (C-C motif) ligand 4 CCL4/MIP1β, Interferon-Gamma IFN-γ, Interleukin-17 IL-17, Interleukin-8 IL-8.

The main findings were firstly that, at univariate analysis, serum concentrations of Il-17 were not associated with IMT. Secondly, circulating concentrations of Il-17 were related to the levels of specific inflammatory/immune regulators, such as TNF α, IL-6, IL-8, IFN *γ,* CCL4/MIP1β, Eotaxin, but not of other inflammatory parameters such as CRP, fibrinogen and ferritin (Table 2). Noteworthy were the strict relationships between serum IL-17 and the parameters strongly up-regulated in allergic/hyperergic reactions, such as eotaxin, IL-8, and CCL4/MIP1β (Table 2, Figure 1). Similarly, IL-8 and eotaxin were the best combination of predictors of their serum levels at multivariate analysis. Of interest, at univariate analysis, IMT was well predicted by the fat deposition in the abdominal, liver and muscle districts, specifically, the thickness of visceral adiposity expressed as VAT, the grade of HS at US, the deposition of TG in muscle, i.e., ImTG, by an IL-17-induced chemokine, i.e., circulating eotaxin, and a marker of chronic inflammation, i.e., serum ferritin, but not by the smoking status (Table 3). Insulin resistance, evaluated as HOMA, and anthropometric features such as BMI, WC and WHR were not associated with serum IL-17 levels, nor were US parameters of ectopic fat storage, such as HS, SAT, VAT and ImTG, related to the same cytokine (Table 3).

**Table 2:** **Predictions of interleukin-17**

	***Coefficient***	***Std. error***	***95% CI***	**t**	**P**
Il-17/WC	−0.073	1.17	−2.40 to 2.26	−0.062	0.95
Il-17/BMI	2.83	2.03	−1.21 to 6.89	1.39	0.16
Il-17/WHR	338.62	314.13	−286.7 to 964.01	0.28	0.28
Il-17 SAT	5.64	21.47	−37.10 to 48.39	0.26	0.79
Il-17/VAT	5.97	6.46	−6.89 to 18.83	0.92	0.35
Il-17/Fibrinogen	0.34	0.21	−0.087 to 0.77	1.58	0.11
Il-17/CRP	2.18	7.61	−12.97 to 17.33	0.28	0.77
Il-17/SLD	−6.54	10.58	−27.61 to 14.52	−0.61	0.53
Il-17/Ferritin	−0.10	0.12	−0.34 to 0.13	−0.84	0.4
Il-17/TNF-α	1.08	0.067	0.94 to 1.21	15.9	<0.0001
Il-17/IL-6	5.15	0.63	3.89 to 6.42	8.1	<0.0001
Il-17/IFN- *γ*	0.44	0.024	0.39 to 0.49	18.0	<0.0001
Il-17/US	9.71	27.96	−45.96 to 65.39	0.34	0.72
Il-17/ALT	−0.71	1.02	−2.75 to 1.31	−0.70	0.48
Il-17/CHE	0.012	0.008	−0.03 to 0.004	−1.47	0.14
Il-17/AP	0.006	0.62	−1.24 to 1.25	0.009	0.99
Il-17/*γ-GT*	0.049	0.81	−1.56 to 1.66	0.061	0.95
Il-17 /IL-8	1.73	0.086	1.55 to 1.90	20.01	<0.0001
Il-17/CXCL10/IP-10	0.0032	0.036	−0.069 to 0.076	0.087	0.93
Il-17/CCL4/MIP1β	3.43	0.45	2.53 to 4.33	7.6	<0.0001
Il-17/Eotaxin	0.78	0.16	0.45 to 1.11	4.73	<0.0001
Il-17/HDL	0.64	1.39	−3.42 to 2.13	−0.46	0.6460
Il-17/LDL	0.12	0.17	−0.21 to 0.45	0.7	0.48
Il-17/HOMA	0.13	4.22	−8.26 to 8.54	0.032	0.97
Il-17/Triglycerides	0.12	0.18	−0.23 to 0.48	0.69	0.48
Il-17/IMT	275.0	485.7	−691.95 to 1241.96	0.56	0.57
Il-17/SBP	0.17	0.43	−0.68 to 1.03	0.41	0.68
Il-17/DBP	−0.35	1.99	−4.32 to 3.60	−0.17	0.85
Il-17/RMR	0.0058	0.038	−0.070 to 0.081	0.15	0.87
Il-17/ImTG	14.80	17.45	−19.94 to 49.5	0.84	0.39

Linear Regression using Eotaxin as depedent variable. Waist Circumference WC, Body Mass Index BMI, Waist to Hip ratio WHR, Subcutaneous Adipose Tissue SAT, Visceral Adipose Tissue VAT, C Reactive Protein CRP, Spleen Longitudinal Diameter SLD, UltraSound US, ALanine aminoTransferase ALT, CHolinEsterase CHE, Alkaline Phosphatase AP, Gamma-Glutamyl Transglutaminase γ-GT, High Density Lipoprotein HDL, Low Density Lipoprotein LDL, HOmeostatic Metabolic Assessment HOMA, Systolic Blood Pressure SPP, Diastolic Blood Pressure DBP, Intima-Media Thickness IMT, Resting Metabolic Rate RMR, C Interferon gamma-induced protein 10 CXCL10/IP-10, Chemokine (C-C motif) ligand 4 CCL4/MIP1β, Interferon-Gamma IFN-γ, Intramuscular Triglyceride (TG) storage Score ImTG, C Interferon Gamma-induced protein 10 CXCL10/IP-10, Chemokine (C-C motif) ligand 4 CCL4/MIP1β, Interferon-Gamma IFN-γ, Tumor Necrosis Factor alpha TNF-α*,* Interleukin-6 IL-6, Interleukin-8 IL-8, Interleukin-17 IL-17.

**Table 3:** **Prediction of carotid intima media thickness by any variable studied**

	***Coefficient***	***Std. error***	***95% CI***	**t**	**P**
IMT/WC	0.00019	0.0002	−0.0003 to 0.0007	0.70	0.48
IMT/BMI	0.00007	0.0004	−0.0008 to 0.001	0.14	0.88
IMT/WHR	0.094	0.072	−0.05 to 0.23	1.3	0.19
IMT/SAT	−0.0079	0.0049	−0.017 to 0.0018	−1.6	0.11
**IMT/VAT**	**0.004**	**0.001**	**0.0016 to 0.007**	**3.1**	**0.0023**
IMT/Fibrinogen	0.00001	0.00005	−0.0001 to 0.00008	−0.29	0.76
IMT/CRP	−0.0007	0.0017	−0.004 to 0.0028	−0.39	0.69
IMT/SLD	−0.0012	0.002	−0.006 to 0.003	−0.5	0.61
**IMT/Ferritin**	**0.00006**	**0.000027**	**0.000005 to 0.0001**	**2.18**	**0.031**
**IMT/HS**	**0.016**	**0.006**	**0.004 to 0.029**	**2.7**	**0.0084**
IMT/ALT	−0.000027	0.0002	−0.0005 to 0.0004	−0.11	0.90
IMT/CHE	−0.0000004	0.000002	−0.000004 to 0.000003	−0.22	0.82
IMT/AP	−0.0002	0.0001	−0.0005 to 0.00005	−1.57	0.11
IMT/*γ-GT*	0.00026	0.00018	−0.0001 to 0.0006	1.39	0.16
IMT/HDL	0.64	1.39	−3.42 to 2.13	−0.46	0.64
IMT/LDL	0.00002	0.00004	−0.00005 to 0.0001	0.66	0.51
IMT/HOMA	0.13	4.22	−8.26 to 8.54	0.032	0.97
IMT/Triglycerides	0.12	0.18	−0.23 to 0.48	0.69	0.48
IMT/Smoking	1.12	1.67	−2.20 to 4.4	0.67	0.50
IMT/SBP	0.0004	0.0002	−0.00008 to 0.0009	1.65	0.10
IMT/DBP	0.00037	0.0004	−0.0005 to 0.0012	0.81	0.41
IMT/RMR	−177.19	1442.46	−3048.9 to 2694.5	−0.12	0.90
**IMT/ImTG**	**0.009**	**0.0039**	**0.0012 to 0.016**	**2.30**	**0.024**
IMT/CXCL10/IP-10	−128.8	1503.1	−3121.47 to 2863.73	−0.085	0.93
IMT/CCL4/MIP1β	0.00004	0.00013	−0.0002 to 0.0003	0.33	0.73
**IMT/Eotaxin**	**705.15**	**281.69**	**144.34 to 1265.95**	**2.50**	**0.014**
IMT/IFN- *γ*	624.95	972.13	−1310.40 to 2560.32	0.64	0.52
IMT/IL-8	39.51	257.14	−472.41 to 551.44	0.15	0.87
IMT/IL-6	27.07	63.77	−99.87 to 154.03	0.42	0.67
IMT/TNF-α	508.12	389.73	−267.77 to 1284.03	1.30	0.19

Linear Regression using IMT as dependent variable. Waist Circumference WC, Body Mass Index BMI, Waist to Hip ratio WHR, Subcutaneous Adipose Tissue SAT, Visceral Adipose Tissue VAT, C Reactive Protein CRP, Spleen Longitudinal Diameter SLD, UltraSound US, ALanine aminoTransferase ALT, CHolinEsterase CHE, Alkaline Phosphatase AP, Gamma-Glutamyl Transglutaminase γ-GT, High Density Lipoprotein HDL, Low Density Lipoprotein LDL, HOmeostatic Metabolic Assessment HOMA, Systolic Blood Pressure SPP, Diastolic Blood Pressure DBP, Intima-Media Thickness IMT, C Interferon Gamma-induced protein 10 CXCL10/IP-10, Chemokine (C-C motif) ligand 4 CCL4/MIP1β, Interferon-Gamma IFN-γ, Tumor Necrosis Factor alpha TNF-α*,* Interleukin-6 IL-6, Interleukin-8 IL-8, Interleukin-17 IL-17. The smoking status was categorized as present/absent.

When the interplay of cytokine/chemokine was evaluated at the multivariate analysis, the best combination of predictors of circulating IL-17 levels were serum IL-8 and serum eotaxin, (Table 4, top). Again, when evaluating the main risk factors for early atherosclerosis at the multivariate analysis, the amount of visceral fat, expressed as VAT, and circulating eotaxin levels were the best combinations in predicting IMT (Table 4, bottom). After adjusting both models for gender, the predictive factors remained unchanged. After adjusting the IMT prediction for other risk factors of atherosclerosis, i.e., adding ***LDL values, HOMA and smoking status, the amount of visceral fat, evaluated by US, and the circulating eotaxin levels in this population, remained predictors***. Obviously, age played a predominant role in the adjusted model, Table 4 (third part).

**Table 4:** **Best combination of cytokines pattern able to predict IL-17 and of factors predicting the intima-media thickness**

**Dependent Y**	**IL-17**				
**Independent variables**	**Coefficient**	**Std. error**	***β***	**t**	**P**
IL-8	1.61	0.085	0.90	18.9	<0.0001
Eotaxin	0.28	0.074	0.39	3.79	0.0003
Variables not included in the model IL-6, TNF-α, CCL4/MIP1β					
***Zero order correlation coefficients:***					
IL-8: r 0.91, IL-6: r 0.67, TNF-α : r 0.87, CCL4/MIP1β: r 0.65, Eotaxin: r 0.47					
Dependent Y	IMT				
Independent variables	Coefficient	Std. Error	*β*	t	P
Eotaxin	0.00011	0.000039	0.30	2.8	0.0059
VAT	0.0046	0.0013	0.36	3.4	0.0010
Variables not included in the model: smoking status ,ferritin, HS grade at US, ImTG score at US, age, LDL,					
***Zero order correlation coefficients:***					
Eotaxin: *r* 0.27, VAT: *r* 0.33, ferritin: *r* 0.24, HS grade at US: *r* 0.29, ImTG score at US: *r* 0.25					
The latter model adjusted for Age					
Dependent Y	IMT				
Independent variables	Coefficient	Std. Error	*β*	t	P
Eotaxin	0.0027	0.000032	0.32	2.8	0.0059
VAT	0.0014	0.0011	0.26	3.4	0.0010
AGE	0.0014	0.00023	0.58	6.20	<0.0001

Zero order correlation coefficients: Eotaxin: r= 0.27, r= VAT: r= 0.33, Age r= 0.61.

Multiple Regression Equations, Stepwise Method*,* using IMT or IL-17 as dependent variables. In blue is evidenced the regression line; in dark red the 95% prediction and in light red the 95% prediction. Chemokine (C-C motif) ligand 4 CCL4/MIP1β, Interferon-Gamma IFN-γ, Interleukin-17 IL-17, Interleukin-8 IL-8 C, Tumor Necrosis Factor alpha TNF-α*,* Interleukin-6 IL-6, Interleukin-17 IL-17.

Surprisingly, serum Il-17 was an independent predictor of IFN-γ levels.

Among the anthropometric measures, i.e., BMI, WHR and WC, only WC was associated only to VAT at US coefficient 0.097; Std. Error0.021; 95% CI 0.46 to 4.57; P = <0.0001.

## Discussion

Evidencing the key-points of this research, firstly, serum Il-17 concentrations were associated with those of IL-6, IFN-*γ* and TNF-α on the one hand (inflammatory cytokines), and to IL-8, CCL4/MIP1β, and eotaxin on the other hand (allergic/hyperergic cytokines, all induced by IL-17). Secondly, early atherosclerosis, evidenced as increased thickness of the arterial intima-media, was strongly predicted, other than by age, by the amount of visceral adiposity, expressed as VAT, and by circulating eotaxin.

The former finding confirms the body of pertinent knowledge that IL-17, induced by IL–23, acts as a potent mediator in delayed-type reactions by increasing cytokines/chemokine production from other cell types i.e., fibroblasts and macrophages beyond endothelial and epithelial cells, the classic model of which being bronchial asthma [27].

The latter finding confirms that an interplay between alterations of adipose tissue distribution and its function with broad effects on cytokine/chemokines in the induction/maintenance of atherosclerosis could be evidenced, although a direct effect of IL-17 on vascular walls was lacking.

Discussing possible mechanisms and explanations for the link between the amount of visceral fat on the one hand, circulating levels of IL-17-related chemokine – i.e., eotaxin as well – and IMT on the other hand, we hypothesize that IL-17, produced by the macrophages of visceral adipose tissue, functioning as immune organ, [28] induces eotaxin secretion by the smooth muscle cells present in the atheromatosus vessels, only via activated cells, after a stimulus by TNF-α [29]. In agreement with our findings on circulating IL-8 levels, it has been recently evidenced that serum concentrations of this cytokine mirror the impaired coronary circulation [30]. This interpretation is supported by the composition of adipose-resident immune cell populations, i.e., macrophages, in obese individuals and animals. In obese individuals, fat cells may act like inflammatory white blood cells by using communication machinery once considered to be peculiar to immune cells. Among others, obesity, is associated with increased IL-17A production in humans [31]. Mirroring this, Th17 cell expansion is observed in obese mice [32]. In response to calorie excess, mice lacking IL-17 exhibit protection from glucose impairment, despite an increased weight gain [33]. Nevertheless, the results of our study are at variance with previously published works proposing an athero-protective role for IL-17 [14,15]. Vice versa, our data are in favor of an indirect pro-atherosclerotic role of IL-17, in agreement with data from animal studies [12,13]. To reinforce this hypothesis, there is a recent finding that IL-17 enhances the production of the von Willebrand factor by human endothelial cells, and induces endothelial cell apoptosis by activating caspase-3 and caspase-9 and up-regulating the ratio of Bax/Bcl-2, suggesting a role for IL-17 in vascular endothelial damage [34]. The role of NAFLD in predicting CAD risk, well established in the literature [35], is further supported by our data. Quite relevant is the finding of the strict correlation between ImTG and IMT, lending credence to the view that ectopic fat storage, also in the muscle, is a sign of insulin resistance [36], resulting central to major cardiovascular events. The link found between ferritin and IMT, is not dampened by the results of a study evaluating the relationship between body iron status and carotid atherosclerosis in middle-aged eastern Finnish men [37], because our estimate was obtained pooling data of both men and women.

A significant role for CXCL10/IP-10 in increasing arterial thickness was not evidenced in our series, differently from previous results [17].

Commenting on IFN-*γ*, our data do not seem to confirm its protective role in early atherosclerosis – expressed as increased carotid intima-media thickness, due to the fact that its circulating levels in our population were not inversely associated with IMT; this finding is in contrast with data from an aforementioned study [14]. To reinforce our line of research, a strong positive relationship between serum IL-17 and serum IFN-*γ* was evidenced.

Coming back to another IL-17-related cytokine, IL-8 induces homing of neutrophils that contribute to myocardial injury [38]. In clinical studies, circulating IL-8 levels were higher in patients with acute myocardial infarction or unstable angina as compared to controls [39], representing a good predictor of CAD [40]. Our data on patients with early atherosclerosis are in support of the aforementioned results. It should be stressed that the behavior of IL-8 changes in the advanced phases of CAD, becoming this cytokine cardio-protective, at least in animal models [41].

As to the limitations of the present study, firstly, the type of study did not allow us to draw conclusions on the direction of the association. In fact, the cross-sectional nature of this design does not fulfil the temporal criteria needed to study the natural history of atherosclerosis. Another possible pitfall was the lack of artery wall biopsy and subsequent culture of myofibroblasts to detect Il-17 tissue expression. Neither was adiposity evaluated by the more precise MRI, nor was arterial stiffness – judged by experts as a marker of asymptomatic atherosclerosis [42] – detected by means of pulse wave velocity. We should mention that crucial future research will be directed towards the study of the TH17/TREG cytokines [43] and human angiogenesis/growth factors, in the context of visceral adiposity, which is reckoned as decisive in predicting CAD [44].

## Conclusion

Our research lends credence to a possible indirect role of IL-17 in determining or favoring early, sub-clinical atherosclerosis in obese patients, in the sense that its circulating levels, strongly linked to those of eotaxin could be the expression of allergic–hyperergic mechanisms triggering this disease.

## References

[1] Fathi R, Marwick TH (2001). Noninvasive tests of vascular function and structure: why and how to perform them. Am Heart J.

[2] Tarantino G, Saldalamacchia G, Conca P, Arena A (2007). Non-alcoholic fatty liver disease: further expression of the metabolic syndrome. J Gastroenterol Hepatol.

[3] Oni ET, Agatston AS, Blaha MJ, Fialkow J, Cury R, Sposito A, Erbel R, Blankstein R, Feldman T, Al-Mallah MH, Santos RD, Budoff MJ, Nasir K (2013). A systematic review: burden and severity of subclinical cardiovascular disease among those with nonalcoholic fatty liver; should we care?. Atherosclerosis.

[4] Novelli EL, Souza GA, Ebaid GM, Rocha KK, Seiva FR, Mani F, Campos KE, Sforcin JM (2010). Energy expenditure and oxygen consumption as novel biomarkers of obesity-induced cardiac disease in rats. Obesity (Silver Spring).

[5] Pillen S, Arts IMP, Zwarts MJ (2008). Muscle ultrasound in neuromuscular disorder. Muscle Nerve.

[6] Therkelsen KE, Pedley A, Speliotes EK, Massaro JM, Murabito J, Hoffmann U, Fox CS (2013). Intramuscular fat and associations with metabolic risk factors in the Framingham Heart Study. Arterioscler Thromb Vasc Biol.

[7] Frostegard J, Ulfgren AK, Nyberg P, Hedin U, Swedenborg J, Andersson U, Hansson GK (1999). Cytokine expression in advanced human atherosclerotic plaques: dominance of pro-inflammatory (th1) and macrophage-stimulating cytokines. Atherosclerosis.

[8] Roselaar SE, Kakkanathu PX, Daugherty A (1996). Lymphocyte populations in atherosclerotic lesions of apoe −/−and ldl receptor −/−mice. Decreasing density with disease progression. Arterioscler Thromb Vasc Biol.

[9] Xu JM, Shi GP (2012). Emerging role of mast cells and macrophages in cardiovascular and metabolic diseases. Endocr Rev.

[10] Tarantino G, Costantini S, Finelli C, Capone F, Guerriro E, La Sala N, Gioia S, Castello G: **Carotid intima-media thickness is predicted by combined eotaxin levels and the severity of hepatic steatosis at ultrasonography in obese patient with NAFLD.** *PLoS One*, in press. 10.1371/journal.pone.0105610 PMC4182088 25268946

[11] Iwakura Y, Ishigame H, Saijo S, Nakae S (2011). Functional specialization of IL-17 family members. Immunity.

[12] Smith E, Prasad KM, Butcher M, Dobrian A, Kolls JK, Ley K, Galkina E (2010). Blockade of interleukin-17A results in reduced atherosclerosis in apolipoprotein E-deficient mice. Circulation.

[13] Gao Q, Jiang Y, Ma T, Zhu F, Gao F, Zhang P, Guo C, Wang Q, Wang X, Ma C, Zhang Y, Chen W, Zhang L (2010). A critical function of Th17 proinflammatory cells in the development of atherosclerotic plaque in mice. J Immunol.

[14] Danzaki K, Matsui Y, Ikesue M, Ohta D, Ito K, Kanayama M, Kurotaki D, Morimoto J, Iwakura Y, Yagita H, Tsutsui H, Uede T (2012). Interleukin-17A deficiency accelerates unstable atherosclerotic plaque formation in apolipoprotein E-deficient mice. Arterioscler Thromb Vasc Biol.

[15] Simon T, Taleb S, Danchin N, Laurans L, Rousseau B, Cattan S, Montely JM, Dubourg O, Tedgui A, Kotti S, Mallat Z (2013). Circulating levels of interleukin-17 and cardiovascular outcomes in patients with acute myocardial infarction. Eur Heart J.

[16] Wang YH, Voo KS, Liu B, Chen CY, Uygungil B, Spoede W, Bernstein JA, Huston DP, Liu YJ (2010). A novel subset of CD4(+) T(H)2 memory/effector cells that produce inflammatory IL-17 cytokine and promote the exacerbation of chronic allergic asthma. J Exp Med.

[17] Rothenbacher D, Müller-Scholze S, Herder C, Koenig W, Kolb H (2006). Differential expression of chemokines, risk of stable coronary heart disease, and correlation with established cardiovascular risk markers. Arterioscler Thromb Vasc Biol.

[18] Montalcini T, Gazzaruso C, Ferro Y, Migliaccio V, Rotundo S, Castagna A, Pujia A (2013). Metabolic fuel utilization and subclinical atherosclerosis in overweight/obese subjects. Endocrine.

[19] Webb M, Yeshua H, Zelber-Sagi S, Santo E, Brazowski E, Halpern Z, Oren R (2009). Diagnostic value of a computerized hepatorenal index for sonographic quantification of liver steatosis. AJR Am J Roentgenol.

[20] Pillen S, van Alfen N (2011). Skeletal muscle ultrasound. Neurol Res.

[21] Heckmatt JZ, Leeman S, Dubowitz V (1982). Ultrasound imaging in the diagnosis of muscle disease. J Pediatr.

[22] Tarantino G, Finelli C, Colao A, Capone D, Tarantino M, Grimaldi E, Chianese D, Gioia S, Pasanisi F, Contaldo F, Scopacasa F, Savastano S (2012). Are hepatic steatosis and carotid intima media thickness associated in obese patients with normal or slightly elevated gamma-glutamyl-transferase?. J Transl Med.

[23] Tarantino G, Marra M, Contaldo F, Pasanisi F (2008). Basal metabolic rate in morbidly obese patients with non-alcoholic fatty liver disease. Clin Invest Med.

[24] Schutz Y, Kyle UU, Pichard C (2002). Fat-free mass index and fat mass index percentiles in Caucasians aged 18–98 y. Int J Obes Relat Metab Disord.

[25] Arellano-Garcia ME, Hu S, Wang J, Henson B, Zhou H, Chia D, Wong DT (2008). Multiplexed immunobead-based assay for detection of oral cancer protein biomarkers in saliva. Oral Dis.

[26] Lédée N, Munaut C, Sérazin V, Perrier d’Hauterive S, Lombardelli L, Logiodice F, Wainer R, Gridelet V, Chaouat G, Frankenne F, Foidart JM, Piccinni MP (2010). Performance evaluation of microbead and ELISA assays for follicular G-CSF: a non-invasive biomarker of oocyte developmental competence for embryo implantation. J Reprod Immunol.

[27] Kawaguchi M, Kokubu F, Fujita J, Huang SK, Hizawa N (2009). Role of interleukin-17F in asthma. Inflamm Allergy Drug Targets.

[28] Makki K, Froguel P, Wolowczuk I (2013). Adipose tissue in obesity-related inflammation and insulin resistance: cells, cytokines, and chemokines. ISRN Inflamm.

[29] Ghaffar O, Hamid Q, Renzi PM, Allakhverdi Z, Molet S, Hogg JC, Shore SA, Luster AD, Lamkhioued B (1999). Constitutive and cytokine-stimulated expression of eotaxin by human airway smooth muscle cells. Am J Respir Crit Care Med.

[30] Brunetti ND, Salvemini G, Cuculo A, Ruggiero A, De Gennaro L, Gaglione A, Di Biase M (2014). Coronary artery ectasia is related to coronary slow flow and inflammatory activation. Atherosclerosis.

[31] Sumarac-Dumanovic M, Stevanovic D, Ljubic A, Jorga J, Simic M, Stamenkovic-Pejkovic D, Starcevic V, Trajkovic V, Micic D (2009). Increased activity of interleukin-23/interleukin-17 proinflammatory axis in obese women. Int J Obes (Lond).

[32] Ahmed M, Gaffen SL (2010). IL-17 in obesity and adipogenesis. Cytokine Growth Factor Rev.

[33] Zuniga LA, Shen WJ, Joyce-Shaikh B, Pyatnova EA, Richards AG, Thom C, Andrade SM, Cua DJ, Kraemer FB, Butcher EC (2010). IL-17 regulates adipogenesis, glucose homeostasis, and obesity. J Immunol.

[34] Zhu F, Wang Q, Guo C, Wang X, Cao X, Shi Y, Gao F, Ma C, Zhang L (2011). IL-17 induces apoptosis of vascular endothelial cells: potential mechanism for human acute coronary syndrome. Clin Immunol.

[35] Chhabra R, O'Keefe JH, Patil H, O'Keefe E, Thompson RC, Ansari S, Kennedy KF, Lee LW, Helzberg JH (2013). Association of coronary artery calcification with hepatic steatosis in asymptomatic individuals. Mayo Clin Proc.

[36] Tarantino G, Caputi A (2011). JNKs, insulin resistance and inflammation: a possible link between NAFLD and coronary artery disease. World J Gastroenterol.

[37] Rauramaa R, Väisänen S, Mercuri M, Rankinen T, Penttila I, Bond MG (1994). Association of risk factors and body iron status to carotid atherosclerosis in middle-aged eastern Finnish men. Eur Heart J.

[38] Riesenberg K, Levy R, Katz A, Galkop S, Schlaeffer F (1997). Neutrophil superoxide release and interleukin 8 in acute myocardial infarction: distinction between complicated and uncomplicated states. Eur J Clin Invest.

[39] Zhou RH, Shi Q, Gao HQ, Shen BJ (2001). Changes in serum interleukin-8 and interleukin-12 levels in patients with ischemic heart disease in a Chinese population. J Atheroscler Thromb.

[40] Boekholdt SM, Peters RJ, Hack CE, Day NE, Luben R, Bingham SA, Wareham NJ, Reitsma PH, Khaw KT (2004). IL-8 plasma concentrations and the risk of future coronary artery disease in apparently healthy men and women: the EPIC-Norfolk prospective population study. Arterioscler Thromb Vasc Biol.

[41] Lefer AM, Johnson G, Ma XL, Tsao PS, Thomas GR (1991). Cardioprotective and endothelial protective effects of [Ala-IL8]77 in a rabbit model of myocardial ischaemia and reperfusion. Br J Pharmacol.

[42] Claridge MW, Bate GR, Hoskins PR, Adam DJ, Bradbury AW, Wilmink AB (2009). Measurement of arterial stiffness in subjects with vascular disease: Are vessel wall changes more sensitive than increase in intima-media thickness?. Atherosclerosis.

[43] Yu XH, Jiang N, Zheng XL, Cayabyab FS, Tang ZB, Tang CK (2014). Interleukin-17A in lipid metabolism and atherosclerosis. Clin Chim Acta.

[44] Savva SC, Lamnisos D, Kafatos AG, Savva SC, Lamnisos D, Kafatos AG (2013). Predicting cardiometabolic risk: waist-to-height ratio or BMI. A meta-analysis. Diabetes Metab Syndr Obes.

